# Editorial: Diabetes and non-alcoholic fatty liver disease: points of physiological and mechanistic intersection and current co-therapeutic approaches

**DOI:** 10.3389/fendo.2023.1253604

**Published:** 2023-07-17

**Authors:** Nick Giannoukakis, Kyle S. McCommis

**Affiliations:** ^1^ Department of Immunology, University of Pittsburgh, Pittsburgh, PA, United States; ^2^ Department of Biochemistry and Molecular Biology, Saint Louis University School of Medicine, St. Louis, MO, United States

**Keywords:** NAFLD, NASH, diabetes, T2D, T1D, pharmacotherapeutics

Non-alcoholic fatty liver disease (NAFLD) has rapidly become the most prevalent liver disease across the globe, with estimates of ~25% of individuals globally having NAFLD (1). The term NAFLD covers a broad spectrum of severity, ranging from “benign” lipid accumulation, often referred to as “simple steatosis”, to non-alcoholic steatohepatitis (NASH) which involves hepatocellular injury, inflammation, and fibrosis. If left untreated, NASH can further progress to cirrhosis, liver failure, hepatocellular carcinoma, and either necessary liver transplantation or death. It should be noted, that as of June 2023, the preferred nomenclature was updated to metabolic dysfunction-associated steatotic liver disease (MASLD) and metabolic dysfunction-associated steatohepatitis (MASH) (2). However, since this Research Topic was initiated well before this nomenclature update, for the purpose of this editorial we will use the NAFLD/NASH nomenclature. However, this updated nomenclature highlights the exact purpose of this special Research Topic.

One of the main factors contributing to the dramatic rise in incidence of NAFLD is its integral connection with obesity, insulin resistance, and diabetes, which are all undergoing their own pandemics. More recently, the mechanistic directionality of these associations has been debated. While hepatic lipid accumulation is believed to contribute directly to whole-body defects in insulin action (3), insulin resistance is also a driving factor for hepatic lipid accumulation due to both excessive adipose tissue lipolysis and increased hepatic *de novo* lipogenesis (4–6). With these controversies in mind, the aim of this special Research Topic was to share research findings in the areas of either type 1 diabetes (T1D) or type 2 diabetes (T2D) related to NAFLD. Additionally, since there are currently no approved therapies for NAFLD, and due to the close relationship between insulin sensitivity and NAFLD, research on therapeutics to treat both diseases concomitantly were also encouraged.

A review article by Memaj and Jornayvaz summarized the current knowledge of the prevalence and pathophysiology of NAFLD in T1D which is much less understood compared to insulin resistance/T2D. This review concluded that NAFLD is more prevalent in T1D subjects compared to the general population, however, notes the difficulty in comparing studies with different criteria for determining NAFLD. This article also notes interesting pathophysiological mechanisms which could drive NAFLD in T1D subjects such as altered insulin delivery and hepatic clearance, as well as noting the association between poor glycemic control and the risk of NAFLD.

Related to the potential for NAFLD driving T2D, an article by Chen C. et al. reported that NAFLD progression associated with the development of incident diabetes. Similarly, Chen Y. et al. reported that in a large Taiwanese population, the presence of high serum markers of liver injury was significantly associated with development of incident diabetes. Additionally, an article by Li, et al. reported that even lean individuals with NAFLD were more susceptible to development of T2D. In another study of lean NAFLD, Zhu et al. describe that the association of high circulating lipids or lipid ratios and NAFLD risk is true in both obese and lean individuals. Other studies in this special Research Topic investigated more specific aspects of diabetes and the role NAFLD may play in the association. Basnet et al. describe the presence of high serum uric acid, or hyperuricemia in T2D, and suggest that the prevalence of NAFLD increases the risk of development of diabetes with hyperuricemia. Lastly, studying a cohort of T2D patients, Deravi et al. found that the presence of NAFLD associated with the diabetic microvascular complications such as diabetic neuropathy, nephropathy, and retinopathy. In summary, these studies suggest that the presence of NAFLD is associated with the later development of T2D or worsening of T2D co-morbidities such as hyperuricemia and microvascular disease.

Provided the profound connection between diabetes and NAFLD, a number of articles in this special Research Topic described the therapeutic options for concomitantly treating both diabetes and NAFLD. In a specific population of individuals with both metabolic syndrome-related NAFLD with sarcopenia, Yi et al. noted that physical activity, more-so than dietary factors, was key to preventing sarcopenia. Several studies investigated pharmacotherapeutic options for treating NAFLD and diabetes. Two studies investigated the potential of incretin-related therapies to improve NAFLD. Tan et al. performed a prospective analysis in T2D subjects treated with the glucagon-like peptide-1 receptor agonist (GLP1-RA), liraglutide, and report that liraglutide use decreased hepatic fibrosis in these T2D subjects. Wang X. et al. performed a prospective study on the use of the dipeptidyl peptidase-4 inhibitor, sitagliptin, and reported that while sitagliptin improved glucose metabolic parameters, there was no significant improvement in hepatic fat content. Yan et al. also discuss the efficacy of GLP1-RAs and compare to the effects of sodium-glucose cotransporter-2 inhibitors (SGLT2i) which alternatively reduce glycemia by preventing renal glucose reabsorption. In this systematic review and meta-analysis, the authors describe that in NAFLD patients, only GLP1-RAs improve markers of insulin resistance, while SGLT2i did not significantly reduce fasting glycemia or insulin resistance. Wang Z. et al. performed a meta-analysis of studies regarding the treatment of NAFLD with the thiazolidinedione insulin sensitizer pioglitazone in patients with and without T2D. This analysis concluded that pioglitazone improved insulin resistance and plasma lipids, and also improved NAFLD in both subjects with and without T2D. Conversely, Huang et al. studied T2D subjects treated with or without metformin, and report that long-term metformin use may actually increase susceptibility to developing NAFLD. Lastly, a review article by Niranjan, et al. summarized the therapeutic options for improving hepatic insulin sensitivity to treat NAFLD, including the potential importance of anti-inflammatory agents. Altogether, these studies suggest that agents that improve insulin action, are also associated with improved NAFLD.

The sole “basic” research study published within this Research Topic was performed by Wu et al. In this study, livers from high-fat diet-fed mice with or without overexpression of the G0/G1 switch gene (G0S2) were subjected to proteomics analysis. G0S2 overexpression led to the differential expression of 125 proteins in these livers, with pathway analysis indicating that G0S2 disrupts the “response to insulin”, which is supported by decreased glucose tolerance and insulin tolerance in these mice. Overall, the authors suggest that G0S2 should be considered a potential target for the treatment of diabetes and NAFLD.

This interesting Research Topic certainly highlights the strong connection between diabetes and NAFLD. With the ongoing pandemics of obesity, diabetes, and NAFLD, research on the vital connections between these diseases will only continue to rise. Additionally, in-depth studies and reviews on therapeutic options to concomitantly treat both diabetes and NAFLD will be of utmost importance due to the current lack of approved treatments for NAFLD. Articles from this Research Topic suggest that therapeutic agents that improve insulin sensitivity associate with NAFLD improvements, whereas agents that may only improve glycemia do not improve NAFLD.

## Author contributions

All authors listed have made a substantial, direct, and intellectual contribution to the work and approved it for publication.
